# Two Cases of Advanced Hepatocellular Carcinoma Who Responded Well to the Combination of Durvalumab Plus Tremelimumab After Disease Progression During Atezolizumab Plus Bevacizumab Therapy Under Bevacizumab Withdrawal

**DOI:** 10.7759/cureus.45385

**Published:** 2023-09-16

**Authors:** Yusuke Kawamura, Norio Akuta, Shunichiro Fujiyama, Fumitaka Suzuki, Hiromitsu Kumada

**Affiliations:** 1 Hepatology, Toranomon Hospital, Tokyo, JPN

**Keywords:** systemic therapy, bevacizumab withdrawal, durvalumab plus tremelimumab, atezolizumab plus bevacizumab, advanced hepatocellular carcinoma

## Abstract

Many systemic chemotherapies, including immune checkpoint inhibitors (ICI), are now available for the treatment of advanced hepatocellular carcinoma. On the other hand, it is often difficult to continue administration of angiogenesis inhibitors in these patients due to various side effects. In the two cases described in this paper, following the introduction of combination therapy with atezolizumab plus bevacizumab (Atezo/Bev), it was difficult to continue bevacizumab treatment due to side effects, such as proteinuria and fluid retention, with disease control in the two patients being ultimately poor. However, both patients experienced treatment success after switching Atezo/Bev to a regimen that included durvalumab, an anti-programmed cell death ligand 1 antibody (anti-PD-L1 antibody) similar to atezolizumab, plus tremelimumab, an anti-cytotoxic T lymphocyte-associated antigen 4 antibody (anti-CTLA-4 antibody) in situations where the continuation of bevacizumab was difficult. The efficacy of subsequent drug sequencing from ICI to another ICI after atezolizumab plus bevacizumab, which is the standard first-line treatment in advanced hepatocellular carcinoma, has not yet been established. We consider that the two cases described in this paper provide valuable information worthy of the report.

## Introduction

The immune checkpoint inhibitor (ICI), atezolizumab plus the molecularly targeted agent (MTA), bevacizumab is recommended as a first-line combination therapy (Atezo/Bev) for the treatment of unresectable, advanced hepatocellular carcinoma (HCC) in current treatment strategies [1]. Although approximately one-third of HCC patients achieve an objective response with Atezo/Bev [2], many cases eventually experience disease progression during the treatment course, necessitating a change in treatment modalities. However, there is currently no established regimen for systemic drug therapy after atezolizumab plus bevacizumab combination therapy, with the choice depending on the condition of each patient [1].

In addition, the combination of durvalumab plus tremelimumab (STRID; single tremelimumab regular interval durvalumab) has recently been approved and made available for the treatment of unresectable advanced hepatocellular carcinoma as a new first-line treatment despite its use beyond second-line treatment not yet fully documented [3].

Durvalumab plus tremelimumab is an immunotherapy that uses anti-PD-L1 and anti-CTLA-4 antibodies without anti-VEGF antibodies, which is expected to be useful for patients who are unable to receive or continue anti-VEGF therapies due to the risk of gastrointestinal bleeding or side effects, such as proteinuria, which are known to be associated with anti-VEGF therapies [4].

However, the efficacy of the treatment sequence from immune checkpoint inhibitors (ICI) to ICI has not been established in the systemic chemotherapy currently used to treat advanced HCC.

In the present study, two patients experienced treatment success after switching from Atezo/Bev to a regimen that included durvalumab, an anti-PD-L1 antibody similar to atezolizumab, plus tremelimumab, an anti-CTLA-4 antibody, under the difficult conditions of continuing bevacizumab. The efficacy of subsequent drug sequencing from ICI to ICI after Atezo/Bev, which is the standard first-line treatment in advanced HCC, has not yet been established, and we consider that the two cases described in this paper are valuable and worthy of reporting.

## Case presentation

**Figure 1:**
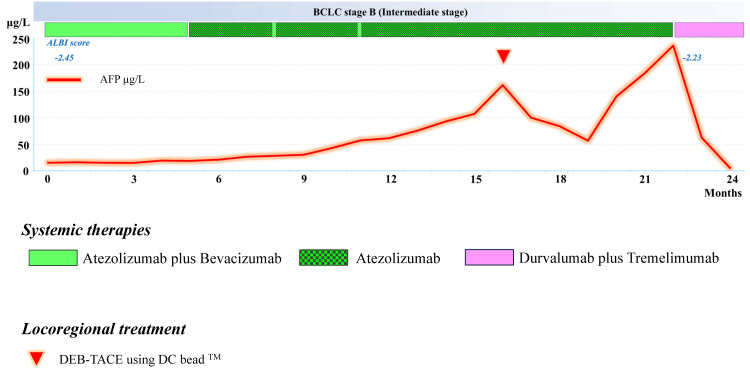
Overall treatment course in Case 1 after the introduction of systemic therapy for unresectable advanced hepatocellular carcinoma *BCLC: Barcelona Clinic Liver Cancer, DEB: drug-eluting beads, ALBI: albumin-bilirubin, TACE: transarterial chemoembolization

Figure 1 shows the treatment course of Case 1, a 77-year-old man, performance status (PS) 0 with Barcelona Clinic Liver Cancer (BCLC) stage B at the time of the introduction of Atezo/Bev (atezolizumab 1200 mg/body and bevacizumab 15 mg/kg every three weeks).

He was treated with this combination of Atezo/Bev for unresectable multiple HCC caused by cirrhosis due to hepatitis C after previous transarterial chemoembolization (TACE) regimens failed to control the disease twice. After the introduction of Atezo/Bev combination therapy, an increase in proteinuria (Common Terminology Criteria for Adverse Events (CTCAE) ver. 5.0 grade 3) was observed, and accordingly, bevacizumab was withdrawn from the fifth month of treatment, and atezolizumab alone was administered. Thereafter, bevacizumab was added at months 8 and 11 when proteinuria decreased, although continued treatment was not possible.

During bevacizumab withdrawal, his alpha-fetoprotein (AFP) level increased gradually and MRI imaging showed suspicious new lesions in the hepatobiliary phase (HBP). Drug-eluting beads (DEB)-TACE using DEB (DC Beads^TM^, Boston Scientific, Marlborough, MA) loaded with epirubicin were administered when his AFP level began to increase rapidly. 

**Figure 2:**
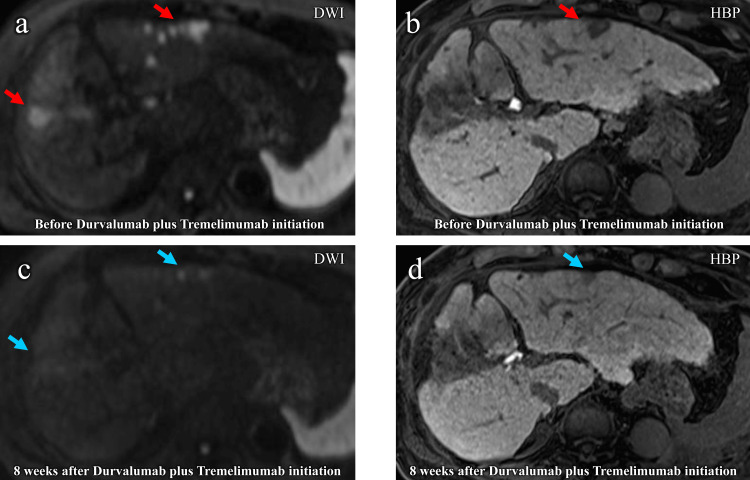
Image changes of MRI images before and after durvalumab plus tremelimumab initiation in Case 1 (a, b) are the diffusion-weighted image (DWI) and hepatobiliary phase (HBP) image before durvalumab plus tremelimumab initiation (red arrows indicate viable tumor); (c, d) are DWI and HBP images eight weeks after durvalumab plus tremelimumab initiation (blue arrows indicate shrunken viable tumor).

Subsequently, the AFP level showed a decreasing trend but increased rapidly again, and the lesion in the area where DEB-TACE was performed also increased. The patient was therefore switched to the combination of Atezo/Bev followed by durvalumab plus tremelimumab (durvalumab 1500 mg/body every four weeks and a single dose of tremelimumab 300 mg/body administered only at induction of therapy according to the STRIDE (single tremelimumab regular interval durvalumab) regimen) at 22 months after the introduction of the Atezo/Bev regimen. Immediately after treatment, an extremely rapid decrease in his AFP level was observed, and in the third month after switching treatment, the AFP level had normalized, with an MRI showing a marked reduction in tumor size and the presence of a partial response (PR) state in response evaluation criteria in solid tumors version 1.1 (RECIST 1.1) (Figure 2) [5].

The patient discontinued treatment after normalization of his AFP level, is being followed up closely, and has had no apparent immune-mediated adverse events (imAEs) during the treatment course, apart from the development of a cardiogenic cerebral stroke related to preexisting atrial fibrillation, which has not adversely affected his daily life.

**Figure 3:**
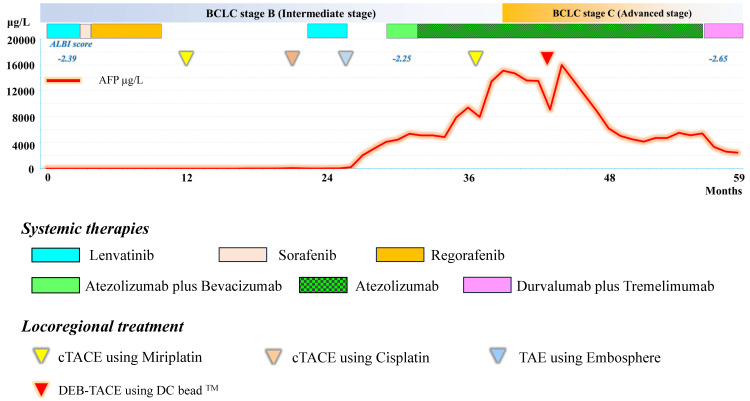
Overall treatment course in Case 2 after introduction of systemic therapy for unresectable advanced hepatocellular carcinoma *BCLC: Barcelona Clinic Liver Cancer, cTACE: conventional transarterial chemoembolization, DEB: drug-eluting beads, ALBI: albumin-bilirubin, TACE: transarterial chemoembolization, TAE: transarterial embolization

Figure 3 shows the treatment course of Case 2, a 69-year-old man, PS 0 with BCLC stage B with chronic kidney disease (CKD) stage 3 renal dysfunction (estimated glomerular filtration rate (eGFR) 41.1 mL/min/1.73 m^2^) at the time of Atezo/Bev introduction.

He had unresectable advanced HCC with alcoholic cirrhosis, which had been difficult to control despite multiple previous TACE procedures, and was then treated with lenvatinib, sorafenib, and regorafenib, followed by additional conventional TACE (cTACE) during the approximately two-year course before the introduction of Atezo/Bev combination therapy. Although the disease was controllable temporarily by TACE, it progressed again, despite performing combination treatment with lenvatinib and transarterial embolization with a rapid increase in AFP level. These findings led to the introduction of Atezo/Bev combination therapy.

Before the introduction of atezolizumab and bevacizumab, an ^18^F-fluorodeoxyglucose positron emission tomography/computed tomography (^18^F-FDG-PET/CT) scan showed no extrahepatic metastases. The patient also had CKD stage 4 renal dysfunction (eGFR 21.0 mL/min/1.73m^2^) at the time of induction.

After induction of Atezo/Bev combination therapy (atezolizumab 1200 mg/ body and bevacizumab 15 mg/kg every three weeks), a fluid retention tendency was observed, and accordingly, bevacizumab was withdrawn. During this withdrawal, rupture of solitary varices in the duodenum was observed, and hemostasis was performed using endoscopic variceal ligation (EVL) and balloon occluded retrograde transvenous obliteration (BRTO). Despite improvement in fluid retention after bevacizumab withdrawal, it was difficult to restart bevacizumab, and treatment was continued with atezolizumab alone. However, the patient’s AFP level showed a gradually increasing trend, and cTACE was performed to control intrahepatic disease progression, although a subsequent chest CT revealed multiple lung metastases and progression of the disease to BCLC stage C.

**Figure 4:**
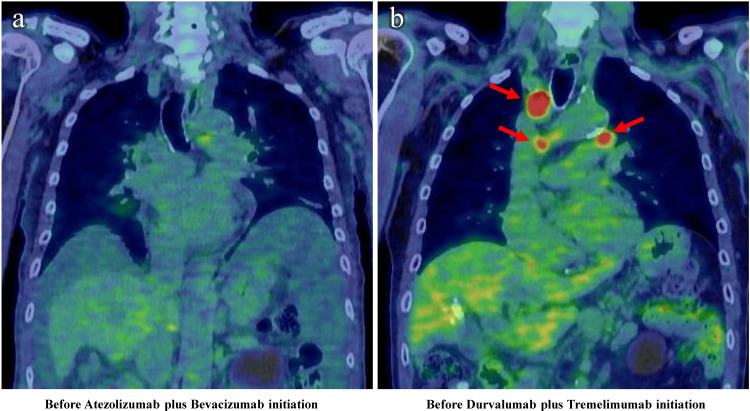
Image changes of 18F-fluorodeoxyglucose positron emission tomography/computed tomography (18F-FDG-PET/CT) during the treatment course in Case 2. (a) is ^18^F-FDG-PET/CT image before atezolizumab plus bevacizumab initiation, and there is no extrahepatic spread observed; (b) is ^18^F-FDG-PET/CT image before durvalumab plus tremelimumab initiation, and there are some extrahepatic spread observed (red arrows indicate mediastinal lymph node metastasis)

As a consequence of this progression, the patient received DEB-TACE using DEB loaded with epirubicin for intrahepatic disease control. After DEB-TACE, AFP was considered to be difficult to control based on the AFP trend, but after DEB-TACE, AFP started to decline slightly later and continued to decline for the next six months. The intrahepatic lesions were also found to be markedly reduced and decreased, although the AFP levels again showed an increasing trend. ^18^F-FDG-PET/CT was therefore performed, which showed new mediastinal lymph node and lung metastases, indicating disease progression (Figure 4).

These findings led to the introduction of durvalumab plus tremelimumab (durvalumab 1500 mg/kg body weight every four weeks and a single dose of tremelimumab 300 mg/kg body weight only at induction of therapy according to the STRIDE regimen).

**Figure 5:**
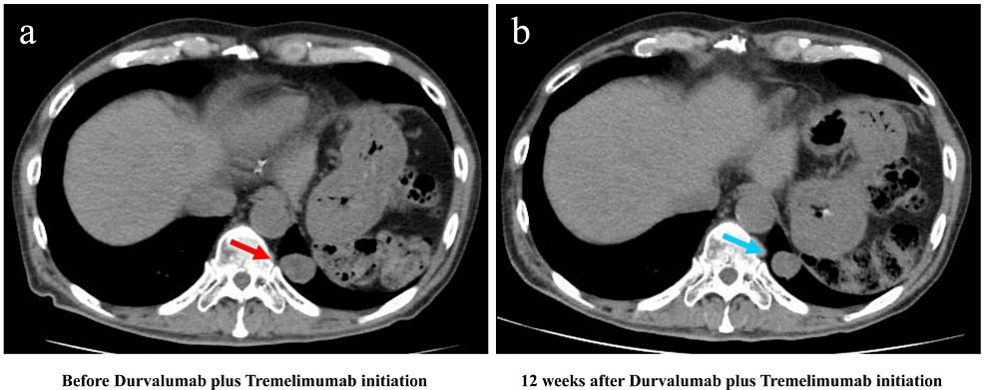
Image changes in CT before and after durvalumab plus tremelimumab initiation in Case 2 (a) CT image before durvalumab plus tremelimumab initiation (red arrow indicates lung metastasis); (b) CT image 12 weeks after durvalumab plus tremelimumab initiation (blue arrow indicates slightly shrunken lung metastasis)

After the introduction of durvalumab plus tremelimumab, the patient’s AFP level showed a decreasing trend, and the extrahepatic metastatic lesions showed a stable disease state in RECIST 1.1 (Figure 5).

The patient is currently on durvalumab monotherapy every four weeks along with the STRIDE regimen without apparent imAEs.

## Discussion

In Japan, the Hepatoma Registry of Integrating and Aggregating Electric Health Records (HERITAGE) study recently reported that lenvatinib has been introduced as a second-line therapy in many patients whose disease had worsened after Atezo/Bev combination therapy [6]. Lenvatinib shows excellent synergy in combination with interventional radiology (IVR), and many real clinical data and clinical trial results have been reported as sequencing from first-line therapy [7-11]. It has also been reported that the combination of drug sequencing and local therapy, including LEN-IVR, is important for prolonging prognosis in patients with advanced liver cancer such as BCLC stage C with intrahepatic target lesions [12].

On the other hand, it is important to maintain hepatic reserve when performing drug sequencing, and it has been reported that an increase in the number of drug sequences leads to overall survival prolongation [13]. Although durvalumab plus tremelimumab has a high imAE rate that requires high-dose steroid use [3], there are high hopes for the drug sequence of immunotherapy to another immunotherapy regime with less impact on the liver reserve. This is especially relevant for patients who are unable to continue anti-VEGF therapy and accordingly, there is an urgent need to verify the effect of these changes in their immunotherapy regime.

This paper describes two cases of disease progression in which continued bevacizumab administration was difficult due to side effects, even after the introduction of Atezo/Bev, with disease control achieved only after switching to durvalumab plus tremelimumab. In both cases, the treatment effect was greater than expected, as the patients were likely to have received the best supportive care under the conventional treatment sequence.

The efficacy of drug sequencing from ICI to ICI with regimens including Atezo/Bev has been reported previously [14], with the response rate for nivolumab plus ipilimumab reported to be 16-30% in RECIST 1.1 and the rate of immune-related adverse events required systemic corticosteroid treatment reported to be 10-12% that included the Atezo/Bev regimen [15-17]. Tremelimumab, which has the same CTLA-4 inhibitory effect as ipilimumab, is also expected to be effective and safe. However, the data of Atezo/Bev to the durvalumab plus tremelimumab sequence has not been reported.

In addition, interestingly, both patients were treated with DEB-TACE before the introduction of durvalumab plus tremelimumab. It is known that DEB releases anticancer drugs gradually over a week or more [18]. It is speculative that, in combination with ICI and DEB-TACE, in addition to tumor antigen release associated with tumor necrosis immediately after embolization, a sustained antigen release from the target lesion may also occur, enhancing the effect of ICI. Therefore, we consider it undeniable that this background may have influenced the therapeutic impact of durvalumab plus tremelimumab in these two cases.

As this study was based on only two cases, we consider it necessary to collect more cases in the future to determine the reproducibility of this study. In addition, durvalumab plus tremelimumab has been introduced as a later-line treatment in nine cases to date, although in the two cases for which imaging evaluation was possible, excluding the two cases presented in this report, the patients were switched to durvalumab plus tremelimumab after continued bevacizumab treatment. In both these cases, disease progression was observed following the initial imaging evaluation, and the patients were therefore switched to other treatments. As durvalumab plus tremelimumab treatment is immunotherapy alone and does not inhibit VEGF, we consider that patients should be carefully monitored for rapid disease progression during the drug sequence from Atezo/Bev to durvalumab plus tremelimumab. On the other hand, future data on durvalumab plus tremelimumab in patients with difficult-to-control proteinuria after tyrosine kinase inhibitors are also expected. We await further reports from multiple centers to determine the extent to which this effect is observed in real-world clinical practice.

## Conclusions

We report our experience with two patients who were treated successfully by switching from atezolizumab to durvalumab plus tremelimumab in situations where continuation of bevacizumab was difficult.
